# Using the Taxonomy and the Metrics: What to Study When and Why: Comment on "Metrics and Evaluation Tools for Patient Engagement in Healthcare Organization- and System-Level Decision-Making: A Systematic Review"

**DOI:** 10.15171/ijhpm.2018.99

**Published:** 2018-10-28

**Authors:** Shoshanna Sofaer

**Affiliations:** ^1^American Institutes for Research, Washington, DC, USA.; ^2^Graduate School of Public Health and Health Policy, City University of New York, New York City, NY, USA.

**Keywords:** Patient Engagement, Program Evaluation, Evaluation Metrics, Logic Model, Patient Participation

## Abstract

Dukhanin and colleagues’ taxonomy of metrics for patient engagement at the organizational and system levels has great potential for supporting more careful and useful evaluations of this ever-growing phenomenon. This commentary highlights the central importance to the taxonomy of metrics assessing the extent of meaningful participation in decision-making by patients, consumers and community members; discusses how the purpose of an evaluation and the organizational relationships among key evaluation stakeholders is likely to influence the choice of metrics in important ways; and suggests a recasting of the metrics in the form of a logic model that supports the selection of metrics that are appropriate for a program given its stage of development and the purposes of the study

This commentary is designed to increase the value and utility of the Dukhanin et al^1^ taxonomy and associated tools, for those who are funding, planning or conducting an evaluation of efforts to engage patients in healthcare organizational, community or system level decision-making. To begin, it is important to recognize that at the core of this study is a commitment by the authors to the idea of meaningful participation in decision-making by individuals who are served by healthcare organizations. The authors’ use of Sherry Arnstein’s “ladder of participation” is a key indicator of this focus.^2^ Arnstein’s “ladder” was produced during the 1960s, when community participation in, indeed control of, decisions about programs intended to improve the circumstances of poor and otherwise vulnerable populations was a widely held goal of those funding and implementing these programs. The authors’ commitment is important because attempts at “engagement” can be designed not to empower, but to effectively co-opt those served by programs. I see little reason to believe that current attempts at patient engagement are immune to such agendas. Healthcare organizations are far more likely to engage their patients and patients’ family members if they believe this will improve their own circumstances, eg, their attractiveness to patients as a service provider; their reputation in the community; and in particular their financial performance. This needs to be kept in mind in the choice of metrics to evaluate patient engagement efforts at the organizational and system levels.^3^

As an evaluator and teacher of evaluation, I have learned that the first step in designing any evaluation must be to identify its purpose(s).^4^ Why is this evaluation being undertaken? Is it a “symbolic” evaluation, designed to demonstrate the willingness of program implementers to be “accountable” and “transparent” not to mention being aware that the current bandwagon has a large sign on it saying “patient engagement”? Or is it a research study, designed to carefully assess whether a fully implemented innovation does or does not achieve its intended goals, to decide if it should be sustained, replicated or taken to scale? Might it be a more “formative” evaluation whose purpose is to figure out whether and how the program has been implemented as intended, and whether, if changes in program design have been made, that is the result of inadequacy of resources available or careful adaptation to local circumstances? The purpose of an evaluation should drive the metrics used: whether they focus on outcomes, processes, or both; whether they are quantitative, qualitative or a mix; whether they are strong, reliable and valid, but most important, their content/focus. As an evaluator, actually figuring out the true purpose of an evaluation can require serious detective work, given that, for example, no one will admit they are just doing a symbolic evaluation. Further, different stakeholders may have different preferences as to the evaluation’s purpose. Typically, however, whoever funds the evaluation calls that tune, which in turn heavily impacts the choice of metrics.

Indeed, evaluation purposes often reflect the organizational relationship between the program, the program’s funder, the program evaluator, and the funder/selector of the evaluator. These relationships typically determine whose purpose will be paramount and which metrics will be used to assess “success.” Notice that, ironically, this list of key parties has historically failed to include the people and the community the program is trying to serve, unless one of the other actors has already done a really fine job of engagement. The increasingly extensive literature in patient participation in research, work that Dukhanin and colleagues appropriately chose not to examine for this analysis, can actually be useful in increasing the involvement of patients in evaluations of patient engagement efforts.^5,6^ This involvement could, indeed, include the selection of key metrics for the study. This will require evaluators to learn how to do this, and do it well.

What difference do organizational relationships make? Consider, for example, a “third-party” evaluation, in which the program funder also selects and funds the evaluator. Almost always, such an evaluation will include outcome metrics, because funders want to “prove” that they funded something that works. All too often, however, they fail to fund careful examination of the actual implementation of the program, which most often requires use of process metrics, although this is changing as everyone realizes that the description of many programs is at best preliminary since the realities of life tend to ensure that changes will be needed. In the case of evaluations of patient engagement efforts, there is a core conundrum here, that is reflected in the authors’ metrics. Is engagement of sufficient value in itself that strong process metrics should be the focus? Or must it be demonstrated that engagement leads to consequences that have historically been of greater importance to healthcare decision-makers, such as improvements in health or health equity, or reductions in cost or inappropriate utilization of services? Note that Dukhanin and colleagues’ “outcome” metrics actually include many of what they call “internal outcomes,” which could well be viewed as “initial” or “intermediate” outcomes. These metrics include impact on engagement participants’ knowledge, skills, empowerment, satisfaction and trust and even impact on the organization or system itself, such as staff views on engagement, formal organization or system policies or explicit changes to decision-making processes. To this reader, these intermediate outcomes are addressing the question “Did meaningful patient engagement actually take place?” which some would consider equivalent to the question “Was the intervention fully implemented?”

Whether we consider these as outcomes or processes, it is critical that these issues be addressed. Educational evaluator Michael Scriven, decades ago, noted that evaluators have to distinguish between “failures of theory” and “failures of practice.”^7^ In this context, if a healthcare organization did not in fact implement patient engagement in a genuine and complete way, then it is not fair to say patient engagement is useless if it did not achieve what the Dukhanin et al call “external outcomes” such as actual changes in health status and health equity.

Connecting these metrics in a meaningful way can be supported by the development of a robust “logic model” or “theory of change” for patient engagement. A logic model lays out the assumed sequence of events through which a program achieves its goals, moving from inputs through processes to outcomes. Avedis Donabedian was a pioneer in asserting that the quality of medical care could indeed be evaluated systematically rather than simply being reviewed by physicians with the same training as the people delivering the care. He distinguished three levels of evaluation: structure (or in language more common to logic model “inputs”), process, and outcomes.^8^ These levels, and the columns in the logic model, are in fact ways to organize the metrics that can be used in an evaluation, depending on when and where and for what purpose the evaluation is being conducted. Thus, when a program is just starting, it is probably wise to focus on the inputs and initial outcomes rather than the long-term outcomes. The logic model (see Table) derives from the author’s experience in designing, conducting and evaluating patient engagement activities in healthcare organizations, and in constructing logic models. It is primarily populated by the metrics in Dukhanin et al and their organization. It has the capacity to help evaluators organize the Dukhanin et al metrics conceptually and temporally.

**Table:** Logic Model: Evaluating Programs to Engage P2C2 Reps in Healthcare Organizations

**Inputs**	**Initial Processes**	**Eventual Processes**	**Initial Outcomes**	**Intermediate Outcomes**	**Long-term Outcomes**
• Organizational commitment to engagement (including financial and staff resources) • Recruitment of an adequate number of P2C2 reps^a^ who are chosen to, reflect the broader population being served, who are ready to participate and have generally positive attitudes toward engagement Training is provided for staff responsible for engagement • Training is provided for P2C2 reps	• Effective recruitment of P2C2 reps using culturally appropriate methods and media • A broad, jargon-free initial assessment of P2C2 needs and strengths is provided to support P2C2 decision-making • P2C2 reps have control over various aspects of decision-making including agenda setting; time allocation; having defined roles; involvement since first stage of decision-making; and control over meeting minutes • Meetings are regularly held; P2C2 reps are not out-numbered by other participants; reps attend regularly and participate actively • Healthcare organization supports dissemination of the results of engagement (eg, decisions made)	• Decision-making process is transparent and trusted; P2C2 reps participate actively; reps participate equally; they are treated with respect; they can make decisions independently; they are involved throughout all stages of decision-making, including final decisions; they are asked to evaluate the decision process and can make changes in it • The healthcare organization assures follow-up of commitments and their translation into action • All steps in the engagement are tailored to the cultural beliefs and practices of P2C2 reps	• Increase in P2C2 reps readiness for and positive attitudes toward engagement • Increase in the knowledge and relevant skills of P2C2 reps • P2C2 reps are satisfied with the engagement; they experience increases in empowerment vis a vis healthcare and increases in their own trust of the healthcare organization • Staff views of engagement become more positive • Explicit changes are made to organization or system process of decision-making • Formal (written) organizational or system policies are developed • The availability of funding and other resources increases^b^	• Healthcare organization experiences improvements in efficiency and cost-effectiveness of their services; service availability, quality and safety; service responsiveness to patient and community needs; and more appropriate use of services^c^ • Additional connections or partnerships are developed with other groups and organizations • Organization or system becomes more accountable to P2C2 they serve • The visibility of the organization or system increases	• Relevant publics become more aware or knowledgeable about health issues • Relevant publics and community gives great support to the organization or system • Population health improves • Health inequalities are reduced • Overall, the P2C2 effort is cost-effective

^a^P2C2 stands for Patient, Public, Consumer and Community. “Reps” stands for representatives. ^b^ Unclear if this means external resources coming to health care organization/system or more resources being assigned to P2C2 activities. ^c^ Depending on the situation, this could mean either *more* or *less* use of specific services.

Thus for example, several of the “preconditions for engagement” metrics identified in the Dukhanin et al article could well be considered structural or “input” measures, or “initial process” measures including the number of patients, consumers and community members involved in the engagement program and their ratio to, for example, hospital staff at meetings; the representativeness and accountability of the patients, consumers and community members involved; the training for staff in patient engagement; and the training of the “P2C2 representatives” themselves. I would add, based on my experience studying community coalitions and consumer dominated governing bodies, whether representatives get solid, ongoing support on technical issues, to help create a level playing field in discussions and decision making with highly training professionals.

One advantage of logic models is that they provide a timeline for evaluators, to ensure that they do not focus on certain metrics too early in the process (eg, looking for intermediate outcomes too early) or too late (eg, failing to make sure that key processes are in place prior to examining outcomes). However, logic models can at times oversimplify the process through which complex and subtle efforts such as patient and community engagement are actually implemented. For example, recruiting members to boards or committees is often an ongoing task, reflecting the difficulties faced by people not getting a salary for important work in continuing for long periods of time. This is a metric, therefore, that probably needs to be continually revisited over the course of implementation and evaluation. Furthermore, it is important that these metrics not be reduced to a “check the box” approach to assessment of patient and community engagement. The processes involved are subtle and nuanced; the “scores” should rarely be simply “yes/no” dichotomies.

Hopefully, this alternative presentation of the metrics will be of value to the field and make it easier to use them in the important job of moving the field of patient and community engagement forward, especially at the organizational rather than the individual level.

## Ethical issues

Not applicable.

## Competing interests

Author declares that she has no competing interests.

## Author’s contribution

SS is the single author of the paper.
